# Shouhui Tongbian Capsules alleviate constipation by modulating the gut microbiota–tryptophan metabolism–AhR signaling axis: a mechanistic study

**DOI:** 10.3389/fmicb.2026.1844612

**Published:** 2026-06-30

**Authors:** Yong Liang, Yang Zhang, Yu Shi, Qimeng Zhang, Hongxi Zhang

**Affiliations:** 1 Liaoning University of Traditional Chinese Medicine, Shenyang, Liaoning, China; 2 Third Affiliated Hospital of Liaoning University of Traditional Chinese Medicine, Shenyang, Liaoning, China

**Keywords:** AhR, constipation, gut microbiota, intestinal barrier, Shouhui Tongbian Capsules, tryptophan

## Abstract

**Objective:**

Constipation is a prevalent functional gastrointestinal disorder characterized by impaired intestinal barrier function, often driven by gut microbiota dysbiosis and metabolic disturbances. Shouhui Tongbian Capsules (SHTB), a traditional Chinese medicine formulation, has demonstrated clinical efficacy in constipation management. This study aimed to elucidate the molecular mechanisms underlying SHTB's anti-constipation effects through modulation of the gut microbiota–tryptophan metabolism–aryl hydrocarbon receptor (AhR) signaling axis.

**Methods:**

A loperamide-induced murine constipation model was established to evaluate the therapeutic efficacy of SHTB, as assessed by time to first black stool defecation, fecal pellet count, fecal water content, and small intestinal propulsion rate. Colonic histopathology was examined by hematoxylin and eosin (H&E) and Alcian blue–periodic acid–Schiff (AB-PAS) staining. 16S rRNA gene sequencing and metabolomic profiling were used to characterize gut microbiota composition and metabolite alterations in SHTB-treated constipated mice. Key findings were subsequently validated through fecal microbiota transplantation (FMT) and pharmacological AhR inhibition.

**Results:**

SHTB significantly improved defecation parameters in constipated mice (*P* < 0.001). Mechanistically, SHTB may promote tryptophan metabolic flux through gut microbiota remodeling, particularly through enrichment of *Bifidobacterium* (*P* < 0.05) and *Alistipes* (*P* < 0.01), leading to activation of colonic AhR signaling (*P* < 0.05) and reinforced intestinal barrier integrity. FMT with SHTB-conditioned microbiota restored key bacterial populations and reactivated AhR expression (*P* < 0.05) in recipient mice. Critically, pharmacological blockade of AhR with CH223191 abolished all SHTB-mediated therapeutic benefits, with no significant differences observed between the CH223191 + Lop and CH223191 + Lop + SHTB groups (*P* > 0.05), thereby identifying AhR as an indispensable mediator of SHTB's anti-constipation mechanism.

**Conclusion:**

SHTB alleviates constipation in a murine model by enhancing intestinal barrier integrity through modulation of the gut microbiota–tryptophan metabolism–AhR signaling axis, thereby providing mechanistic support for its clinical application in managing constipation.

## 1 Introduction

Chronic constipation is among the most prevalent functional gastrointestinal disorders encountered in clinical practice, characterized by infrequent defecation, straining, and passage of hard or lumpy stools (Bharucha and Lacy, 2020). Globally, its prevalence ranges from 12 to 19%, increases with age, and disproportionately affects women (Yildirim et al., 2021). The etiology and pathophysiology of constipation are multifactorial, encompassing genetic predisposition, psychosocial influences, dietary habits, gut microbiota dysbiosis, and impaired intestinal motility, in the absence of identifiable organic pathology. This complexity renders accurate diagnosis and effective treatment particularly challenging (Rao et al., 2016). Chronic constipation is associated not only with anorectal complications such as hemorrhoids and anal fissures, but also with significant psychological comorbidities. Severe cases may trigger carcinogenesis, significantly affecting patients' health-related quality of life (Sundbøll et al., 2019; Alburakan et al., 2022). Currently, laxatives and 5-HT receptor agonists are commonly used in clinical practice to promote gastrointestinal motility for treating constipation. However, long-term use of these medications often causes adverse events such as drug dependence and gastric injury (Bharucha et al., 2013). Therefore, identifying therapeutic strategies that effectively alleviate constipation symptoms while minimizing adverse effects represents an important unmet clinical need.

In recent years, the rapid development of metabolomics technology has provided new perspectives for exploring the complex interactions between the gut microbiota and host health/disease (Chen et al., 2024). The gut microbiota of constipation patients shows significant differences compared to that of healthy individuals (Wang et al., 2023). The metabolic characteristics of constipation patients are manifested as abnormalities in amino acid metabolism (such as tryptophan and glycine), lipid metabolism, and changes in gut microbial metabolites like short-chain fatty acids, which are associated with abnormal intestinal motility (Martin et al., 2024). Studies have found that functional constipation patients often exhibit tryptophan metabolism disorders, characterized by reduced levels of 5-hydroxyindoleacetic acid (5-HIAA) in urine and increased levels of kynurenine (Kyn) and 3-indoxyl sulfate (3-IS). Moreover, the severity of constipation is negatively correlated with 5-HIAA levels and positively correlated with 3-IS levels (Blonska et al., 2023). Supplementing L-tryptophan or modulating the gut microbiota with probiotics can increase tryptophan and 5-HIAA levels in the colon, enhance intestinal motility, and improve constipation symptoms (Legan et al., 2023; Chojnacki et al., 2024). Therefore, regulating the gut microbiota to promote the production of tryptophan metabolites such as 5-HIAA may be an effective strategy for alleviating constipation symptoms (Duan et al., 2023; Zheng et al., 2025). In constipation, dry and hard stools can directly damage the intestinal mucosa, leading to reduced expression of tight-junction proteins, increased intestinal permeability, and impaired barrier function (Umamahesan et al., 2025; Wang et al., 2025). Long-term constipation may also trigger intestinal inflammation, further damaging the intestinal barrier and creating a vicious cycle. Indole-like substances derived from tryptophan are natural ligands of the aryl hydrocarbon receptor (AhR). Activation of AhR can regulate immune cells (such as Tregs and Th17) and the production of their cytokines (particularly IL-22), thereby promoting the protection, repair, and homeostasis of the intestinal barrier (Wu et al., 2025). Additionally, some metabolites of the Kyn pathway in tryptophan, such as Kyn, Kynurenic acid (KYNA), and xanthurenic acid (XA), can also act as AhR ligands (Levé et al., 2025). Based on these findings, modulating tryptophan metabolism via the gut microbiota to activate AhR and repair intestinal barrier integrity is an important strategy for preventing and alleviating constipation.

Traditional Chinese Medicine (TCM) offers distinct therapeutic benefits in the management of constipation. Multiple randomized, double-blind, placebo-controlled studies have found that Chinese herbal medicine can effectively alleviate the symptoms of constipation, with safety similar to that of a placebo (Bensoussan et al., 2015; Zhong et al., 2019). Within the framework of TCM, while the large intestine is identified as the principal site of constipation, its pathogenesis is intrinsically linked to the functional states of the spleen, liver, and kidneys. It is often caused by deficiencies in qi, blood, yin, and yang, leading to a deficiency of intestinal fluids. Si Jun Zi Tang, derived from Tai Ping Hui Min He Ji Ju Fang (1148 AD), is a classic formula for replenishing qi and invigorating the spleen. Zhizhu Tang, from Jin Gui Yao Lue (1065 AD), is a formula for promoting qi circulation and resolving masses. Grounded in these theoretical principles, Shouhui Tongbian Capsules (SHTB) integrate components from both classical formulas. Its ingredients include *Citrus*×*aurantium L., Atractylodes macrocephala Koidz., Lycium chinense Mill., Panax ginseng C.A.Mey., Colla Corii Asini*., *Senna tora (L.) Roxb*., *Aloe vera (L.) Burm.f., Reynoutria multiflora (Thunb.) Moldenke*. It has the effects of replenishing qi and nourishing yin, as well as purging turbidity and promoting bowel movements (Zhang et al., 2025). In 2015, the China Food and Drug Administration granted clinical approval to SHTB (National Drug Approval No. Z20150041) as a proprietary Chinese medicinal product; it has since been extensively employed in clinical practice for the management of constipation (Gong et al., 2024). Studies have shown that SHTB significantly improves overall response rates in constipated patients, relieves symptoms including dyschezia, spontaneous bowel movement frequency, and stool consistency, and effectively reduces both adverse reactions and recurrence rates (Guo et al., 2023; Cui et al., 2025). Initial investigations have demonstrated that SHTB modulates the composition and functional profile of the gut microbiota in constipated rat models, thereby enhancing intestinal motility (Yang et al., 2024). SHTB has also been shown to improve heart failure and atrial fibrillation by modulating the gut microbiota (Chen et al., 2025) and to regulate microbial composition to promote calcium absorption and prevent osteoporosis (Xu et al., 2025). Although several studies have reported that SHTB exerts therapeutic effects through modulation of the gut microbiota, the specific mechanisms by which SHTB influences downstream microbial metabolites and related molecules to exert anti-constipation effects remain unclear.

Despite accumulating evidence linking SHTB to gut microbiota modulation, the downstream metabolic and molecular mechanisms through which SHTB exerts its anti-constipation effects remain poorly defined. Specifically, whether SHTB promotes tryptophan-indole metabolism via microbiota remodeling to activate AhR and restore intestinal barrier integrity has not been systematically investigated. To address this gap, the present study employed 16S rRNA sequencing, targeted and non-targeted metabolomics, fecal microbiota transplantation (FMT), and pharmacological AhR inhibition to elucidate the mechanistic cascade underlying SHTB's therapeutic efficacy against constipation.

## 2 Materials and methods

### 2.1 Materials

SHTB (Approval No.: Z20150041; Batch No.: 26220161) was provided by Lunan Pharmaceutical Group (Shandong, China). Loperamide hydrochloride (Lop) capsules (Approval No.: H10910085; Batch No.: NHJ5102) were obtained from Xi'an Janssen Pharmaceutical Co., Ltd. (Xi'an, Shaaxi, China). Mosapride citrate dispersible tablets (Approval No.: H20031110; Batch No.: 4B001) were obtained from Chengdu Kanghong Pharmaceutical Group Co., Ltd. (Chengdu, Sichuan, China). The AhR antagonist CH223191 (C303374) and antibiotic cocktail (ABX) components—ampicillin (A433389), neomycin sulfate (N109017), vancomycin (V301569), and metronidazole (B300250)—were acquired from Shanghai Aladdin Biochemical Co., Ltd. (Shanghai, China). Primary antibodies targeting GAPDH (60004-1-Ig), AhR (67785-1-Ig), CYP1A1 (13241-1-AP), and ZO-1 (82870-7-RR) were purchased from Proteintech Biotechnology Co., Ltd. (Hubei, China). Anti-Occludin antibody (DF7504) was purchased from Affinity Biosciences (Jiangsu, China). RNA extraction kit (TCH020), reverse transcription kit (RR092A), and fluorescent quantitative PCR reagents (RR820A) were purchased from Takara Biomedical Technology Co., Ltd. (Beijing, China).

### 2.2 UPLC-Q-Orbitrap-MS analysis of SHTB

SHTB comprises the following herbal ingredients: *Citrus* × *aurantium L*. (50 g), *Atractylodes macrocephala Koidz*. (50 g), *Colla Corii Asini*. (75 g), *Lycium chinense Mill*. (75 g), *Panax ginseng C.A.Mey*. (50 g), *Senna tora (L.) Roxb*. (140 g), *Aloe vera (L.) Burm.f*. (160 g), and *Reynoutria multiflora (Thunb.) Moldenke*. (120 g). SHTB complies with the Pharmacopeia of the People's Republic of China (2020 edition) and Good Manufacturing Practice standards; all botanical names were verified through the Medicinal Plant Names Services database (MPNS; http://mpns.kew.org). Detailed preparation procedures are provided in Supplementary materials S1, S2.

The metabolites of SHTB were performed by ultra-performance liquid chromatography coupled to a Q-Orbitrap high-resolution mass spectrometer (UPLC–Q-Orbitrap–MS). High-resolution chromatographic and mass spectrometric datasets were processed using Compound Discoverer 3.3 (CD 3.3), and metabolites were identified against the mzCloud spectral repository. Features with an mzCloud match score ≥80 and a mass deviation <5 ppm were designated as the primary metabolites of SHTB (Gao et al., 2025). Identifications were further corroborated by evaluating peak area, retention time, mass-to-charge ratio (m/z), source classification, and published literature. Detailed analytical methods are provided in Supplementary material S2.

### 2.3 Animal treatment

Male C57BL/6 mice (body weight 14–18 g, age 5–6 weeks) were purchased from Liaoning Changsheng Biotechnology Co., Ltd. (Liaoning, China) (License No.: SCXK [Liao] 2020-0001) and housed in the specific-pathogen-free (SPF)-grade animal facility of the Laboratory Animal Center of Liaoning University of Traditional Chinese Medicine (License No.: SYXK [Liao] 2019-0004) under controlled conditions (temperature 22–25 °C, relative humidity 40%−60%, 12-h light/dark cycle) with *ad libitum* access to standard chow and water. All experimental protocols were approved by the Medical Ethics Committee of Liaoning University of Traditional Chinese Medicine (Approval No.: 21000042024029) and conducted in strict accordance with the *Guide for the Care and Use of Laboratory Animals*.

Experiment 1: following a 7-day acclimatization period, mice were randomized into five groups (*n* = 6 per group): control, loperamide (Lop; model), SHTB low-dose (SHTBL, 150 mg/kg), SHTB high-dose (SHTBH, 300 mg/kg), and positive-control mosapride (Mos, 3 mg/kg). From day 0 to 28, all groups except the control received loperamide (10 mg/kg) by oral gavage once daily to establish the constipation model. From day 14 to 28, each group received its respective treatment by gavage 1 h after loperamide administration; the control group received an equivalent volume of sterile saline throughout. SHTB doses were selected based on published literature (Sun et al., 2023).

Experiment 2: after acclimatization, mice were randomized into seven groups (*n* = 6 per group): control, model (Lop), SHTBH, FMT + Lop, FMT + SHTBH, CH223191 + Lop, and CH223191 + Lop + SHTBH. From day 0 to 28, all non-control groups received loperamide (10 mg/kg, gavage) once daily. The CH223191 + Lop and CH223191 + Lop + SHTBH groups additionally received daily intraperitoneal (i.p.) injections of CH223191 (10 mg/kg). From day 0 to 14, mice in the FMT + Lop and FMT + SHTBH groups received an antibiotic cocktail (ABX) in drinking water (ampicillin 1 g/L, neomycin sulfate 1 g/L, metronidazole 1 g/L, vancomycin 500 mg/L) to generate a pseudo-germ-free state; successful microbiome depletion was confirmed by quantitative PCR (qPCR) quantification of fecal 16S rRNA gene copy numbers (Liang et al., 2020). The FMT method referred to previous literature (Bai et al., 2022) with some modifications. Fresh fecal samples collected from model-group and SHTBH-group donor mice were homogenized in sterile phosphate-buffered saline (PBS), centrifuged at 800 × g for 5 min, and filtered through a 200-mesh sieve to prepare bacterial suspensions. From day 14 to 28, FMT + Lop and FMT + SHTBH recipients received 0.2 mL of the corresponding bacterial suspension by oral gavage once daily, while SHTBH and CH223191 + Lop + SHTBH groups received SHTB (300 mg/kg) by gavage.

### 2.4 Sample collection

Mice were euthanized by intraperitoneal injection of 1% sodium pentobarbital solution (150 mg/kg). A 2–3 cm segment of proximal colon tissue, collected approximately 3 cm distal to the cecum, was fixed in 4% paraformaldehyde for subsequent histological analysis. The remaining colonic tissue and fecal samples were immediately snap-frozen in liquid nitrogen and stored at −80 °C until analysis.

### 2.5 Evaluation of the laxative effect of SHTB on constipation mice

#### 2.5.1 Measurement of first black feces defecation time, fecal pellet count, and fecal water content

On day 26, mice were fasted (food withheld) but allowed free access to water for 12 h. On day 27, 30 min after drug administration, 0.5 mL of a 5% activated charcoal suspension was administered by oral gavage. Normal feeding and drinking were then resumed, and mice were housed individually. The time to the appearance of the first black stool was recorded. Feces were collected over the following 6-h period for pellet enumeration. Samples were then dried in an oven at 90 °C for 3 h. The fecal water content (%) was calculated as: [(wet weight – dry weight) / wet weight] × 100%.

#### 2.5.2 Measurement of intestinal transit rate

On day 28, mice were fasted for 12 h before the final drug administration. Thirty minutes after the final gavage, 0.5 mL of a 5% activated charcoal suspension was administered orally. Following euthanasia, the intestinal tract from the pylorus to the rectum was removed and straightened under tension-free conditions, and the small intestinal transit rate was calculated as: Small intestinal transit rate (%) = [distance traveled by the charcoal front (cm) / total length of the small intestine (cm)] × 100%.

### 2.6 Histological examination

Colon tissues were paraffin-embedded, sectioned at 4 μm, deparaffinized, and rehydrated. Histological morphology was assessed by hematoxylin and eosin (H&E) staining and Alcian blue–periodic acid–Schiff (AB–PAS) staining.

### 2.7 Immunofluorescence

Paraffin-embedded colon sections underwent deparaffinization, rehydration, and antigen retrieval in EDTA buffer (pH 9.0). Following 30-min blocking in 3% bovine serum albumin (BSA), sections were incubated overnight at 4 °C with primary antibodies against ZO-1 (1:300) and occludin (1:200). On the following day, slides were equilibrated to room temperature for 30 min and then incubated with fluorophore-conjugated secondary antibodies for 1 h in the dark at room temperature. Nuclear counterstaining was performed with DAPI (10 min), and immunofluorescence signals were visualized by fluorescence microscopy.

### 2.8 Western blot (WB) analysis

Colonic specimens were homogenized in protein lysis buffer and centrifuged to isolate the supernatant. Protein concentration was determined by the bicinchoninic acid (BCA) assay. Aliquots were combined with loading buffer and denatured at 95 °C for 10 min. Proteins (60 μg per lane) were resolved by SDS-PAGE (300 V, 25 min) and transferred to polyvinylidene fluoride (PVDF) membranes by wet transfer (400 mA, 40 min). Membranes were blocked for 15 min, then incubated overnight at 4 °C with primary antibodies against AhR (1:5,000) and CYP1A1 (1:2,000), followed by incubation with horseradish peroxidase (HRP)-conjugated secondary antibodies for 1 h at room temperature. Bands were visualized by chemiluminescence and quantified using ImageJ software (National Institutes of Health, Bethesda, MD, USA).

### 2.9 Reverse transcription–quantitative PCR (RT-qPCR)

Total RNA was isolated from colonic specimens using a commercial extraction kit per the manufacturer's protocol. RNA integrity and purity were assessed by the A260/A280 absorbance ratio. First-strand cDNA was synthesized by reverse transcription, and RT-qPCR was performed using SYBR Green chemistry on a CFX96 detection platform (Bio-Rad, Hercules, CA, USA). Primer sequences are provided in Supplementary material S3.

### 2.10 16S rRNA sequencing

Total microbial genomic DNA was extracted from fecal samples using a fecal genomic DNA extraction kit (AU46111-96, BioTeke, China) per the manufacturer's instructions. DNA concentration was determined by Qubit fluorometry (Invitrogen, USA). The V3–V4 hypervariable regions of the bacterial 16S rRNA gene were amplified using the universal primer pair 341F (5′-CCTACGGGNGGCWGCAG-3′) and 805R (5′-GACTACHVGGGTATCTAATCC-3′). Amplicons were purified with AMPure XT beads (Beckman Coulter Genomics, USA), requantified by Qubit, and quality-assessed on an Agilent 2100 Bioanalyzer and by Kapa Illumina Library Quantification Kit. Paired-end sequencing (2 × 250 bp) was performed on the Illumina NovaSeq 6000 platform by LC-Bio Technology Co., Ltd. (Hangzhou, China). Alpha and beta diversity indices were calculated in QIIME2. Relative abundance was used for taxonomic profiling. Differentially abundant genera were identified by the Mann–Whitney *U* test (*P* < 0.05). Linear discriminant analysis effect size (LEfSe; LDA score ≥3.0, *P* < 0.05) was computed using nsegata-lefse. Additional figures were generated using the R package (v3.4.4). Detailed protocols are provided in Supplementary material S4.

### 2.11 Untargeted metabolomic analysis

Untargeted metabolomics of mouse fecal samples was performed by LC-Bio Technology Co., Ltd. Metabolites were extracted from thawed fecal samples using 50% methanol buffer and analyzed by liquid chromatography–high-resolution mass spectrometry on an UltiMate 3000 UPLC system (Thermo Fisher Scientific, Waltham, MA, USA) coupled to a Q-Exactive mass spectrometer (Thermo Scientific), with reversed-phase separation on an ACQUITY UPLC T3 column (100 mm × 2.1 mm, 1.8 μm; Waters, Milford, MA, USA). Raw data were preprocessed using XCMS software for peak detection, feature grouping, retention time alignment, and isotope/adduct annotation. Metabolite identities were assigned by matching against the KEGG and HMDB databases. Abundance values were log_2_-transformed and analyzed by *t*-test. Differentially abundant metabolites were defined by fold change (FC) ≥ 1.2 or ≤ 1/1.2, *P* < 0.05, and variable importance in projection (VIP) score ≥1. Pathway enrichment analysis of shared differential metabolites was performed using MetaboAnalyst 6.0 (https://www.metaboanalyst.ca/).

### 2.12 Analysis of tryptophan metabolism

Targeted metabolomics of fecal and colonic samples was performed by LC-Bio Technology Co., Ltd. Approximately 50 mg of each sample was weighed, homogenized in 500 μL of 80% aqueous methanol with steel beads, vortex-extracted for 20 min, and centrifuged at 20,000 × g for 15 min at 4 °C. The supernatant was transferred to a 1.5-mL tube, dried under vacuum concentration, and reconstituted in 100 μL of 5 mmol/L ammonium acetate (with 0.01% formic acid)/acetonitrile (95:5, v/v). Quantitative analysis was performed on an AB Sciex Jasper UPLC system coupled to an AB SCIEX 4500MD triple-quadrupole mass spectrometer. Correlation analysis among differential metabolites, differential bacterial genera, and constipation-related endpoints was conducted using MetOrigin 2.0 (http://metorigin.met-bioinformatics.cn; Deep MetOrigin Analysis; host: *Mus musculus*; Mann–Whitney *U* test; *P* < 0.05; Spearman correlation). Detailed protocols are provided in Supplementary materials S5, S6.

### 2.13 Statistical methods

All data are expressed as mean ± standard deviation (SD). Statistical comparisons were performed using one-way or two-way analysis of variance (ANOVA) followed by Bonferroni's *post hoc* test for multiple comparisons. All analyses were conducted with GraphPad Prism 10 software (Dotmatics, GraphPad Software, San Diego, CA, USA). *P* < 0.05 was considered statistically significant.

## 3 Results

### 3.1 Metabolites of SHTB

**Figure 1:**
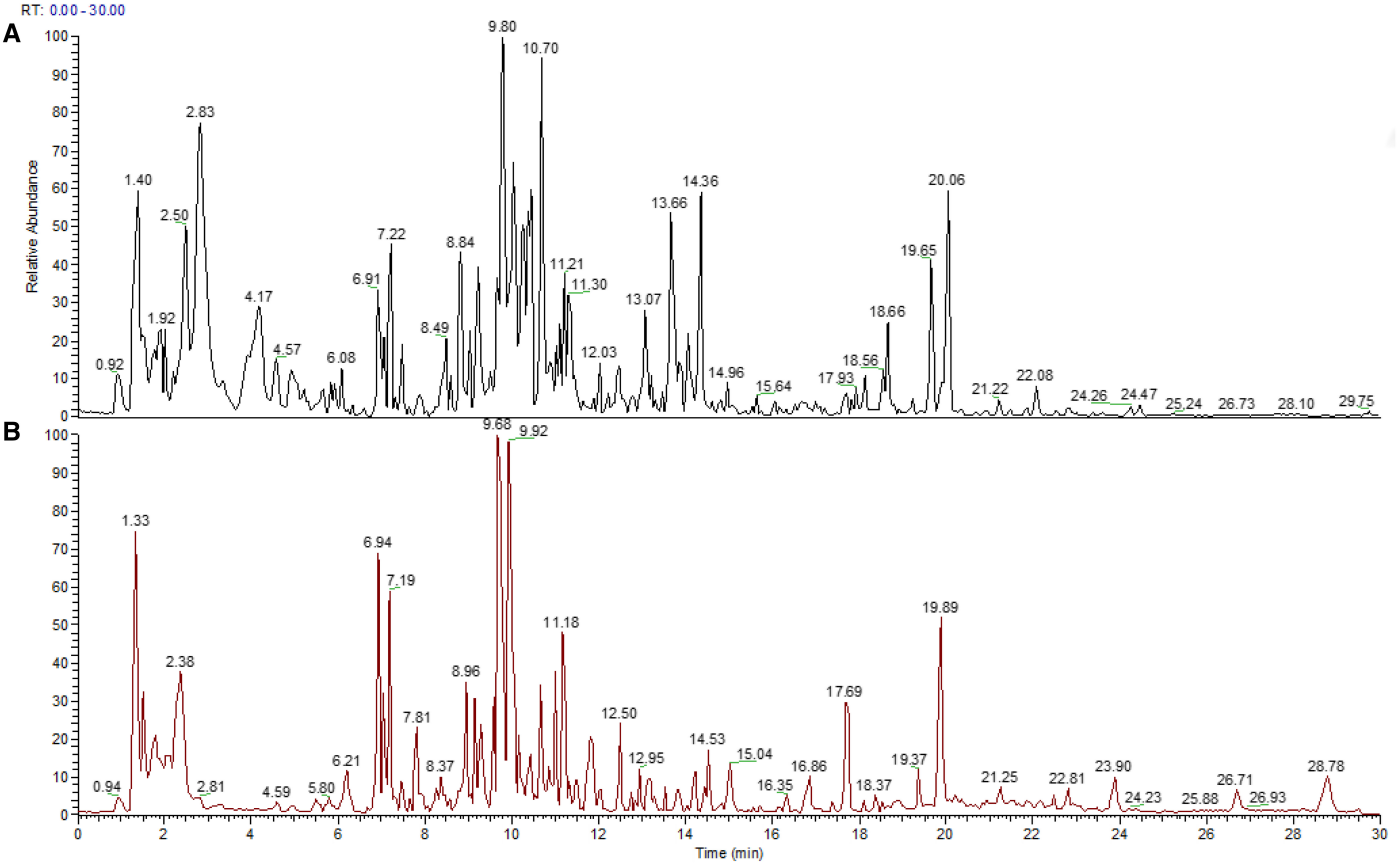
Total ion flow diagram of UPLC-Q-Orbitrap-MS of SHTB in positive ion mode **(A)** and in negative ion mode **(B)**.

The main metabolites of SHTB were characterized by UPLC–Q-Orbitrap–MS analysis. A total of 23 compounds were identified, comprising six coumarins, five isoflavones, two flavonoids, three quinones/ketones, one phenolic acid, one characteristic aloe metabolite, two fatty acids, two amino acids, and one cyclic dipeptide. Total ion chromatograms in positive- and negative-ion modes are presented in Figure 1. Detailed metabolites information is provided in Supplementary Table S7.

### 3.2 SHTB improves defecation functions in constipated mice

**Figure 2:**
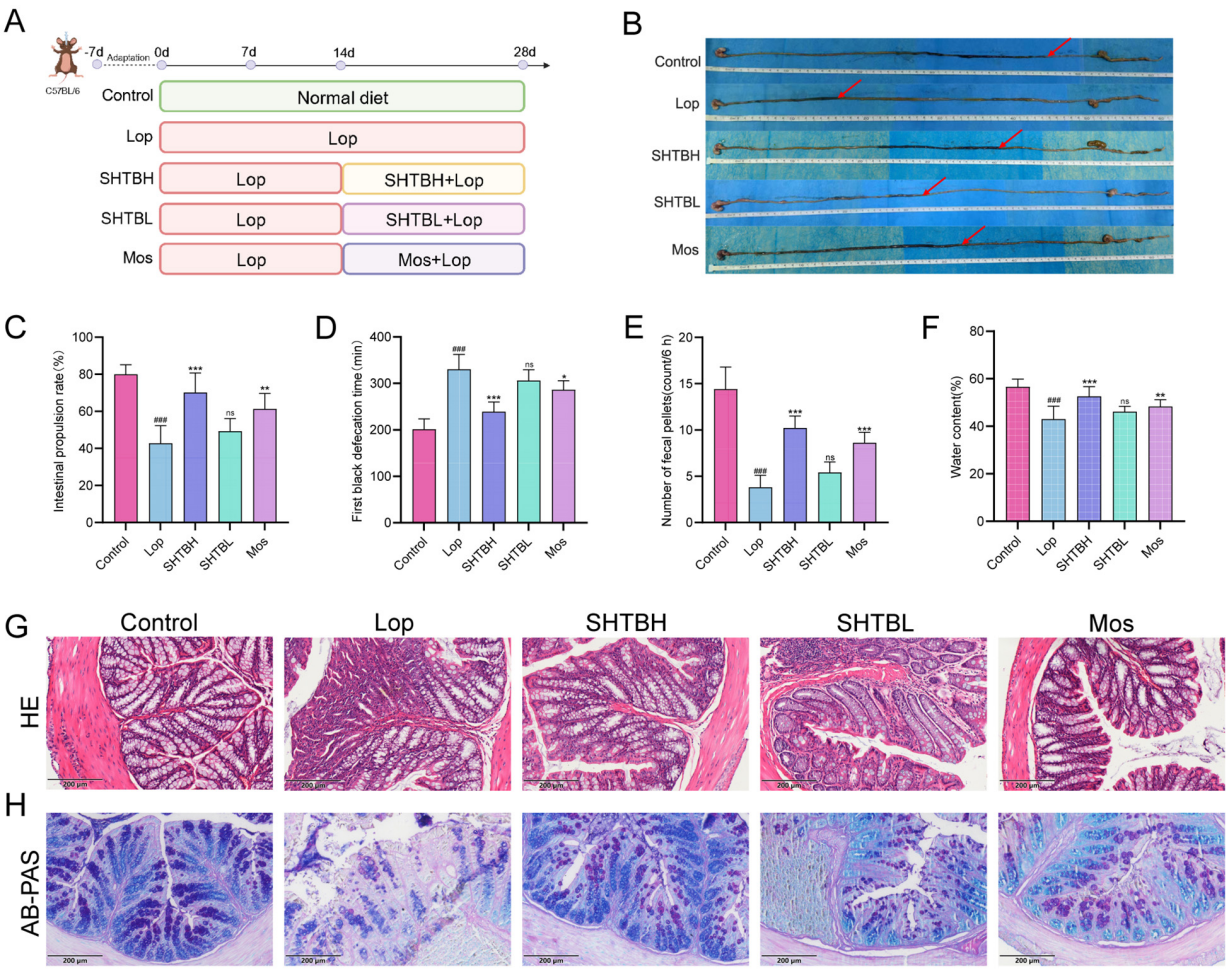
SHTB ameliorates defecation function in constipated mice. **(A)** Schematic diagram of the experimental design. **(B)** Representative images of intestinal propulsion. **(C)** Intestinal propulsion rate; **(D)** First black stool defecation time. **(E)** Number of fecal pellets expelled within 6 h. **(F)** fecal water content. **(G)** Hematoxylin and eosin (HE) staining of mouse colon (Bar = 200 μm). **(H)** Alcian blue–periodic acid–Schiff (AB-PAS) staining of mouse colon (Bar=200 μm). Data are presented as mean ± SD. Compared with the control group: ^#^*P* < 0.05, ^##^*P* < 0.01, ^###^*P* < 0.001; compared with the Lop group: *P < 0.05, **P < 0.01, ***P < 0.001; *n* =6.

As shown in Figure 2, loperamide-treated mice exhibited dyschezia compared to controls, a prolonged latency to the first black stool excretion, a significantly reduced number of fecal pellets and fecal water content within 6 h, and a significantly decreased small intestinal propulsion rate. Treatment with SHTBH and Mos significantly improved the defecatory function of mice (Figures 2B–F). The results of HE and AB-PAS staining showed that the colon tissue structure of mice in the control group was intact without obvious pathological changes, while the colon tissue structure of mice in the Lop group exhibited structural damage characterized by crypt distortion, accompanied by mild inflammatory infiltration and a reduction in goblet cells. After SHTB treatment, the pathological changes in colon tissue and the number of goblet cells significantly improved, most notably in the SHTBH group (Figure 2G). These results indicate that SHTB can alleviate constipation-related symptoms.

### 3.3 SHTB alleviates gut microbiota dysbiosis in constipated mice

**Figure 3:**
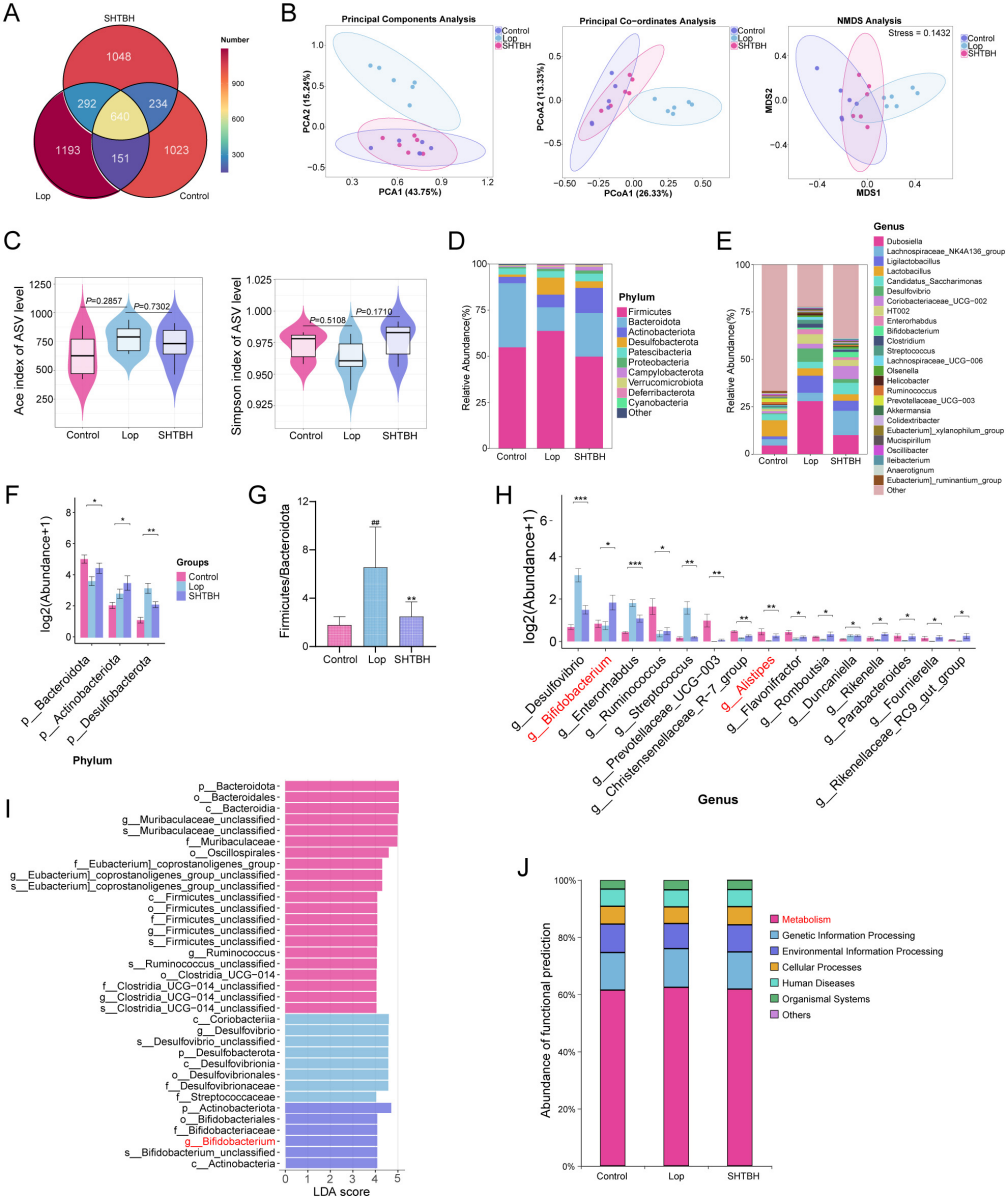
SHTB ameliorates intestinal dysbiosis in constipated mice. **(A)** Venn diagram of ASV distribution. **(B)** Beta diversity analysis based on PCA, PCoA, and NMDS. **(C)** Comparison of Alpha diversity indices (Ace, Chao, Simpson) among groups. **(D)** Stacked bar plot of species abundance at the phylum level. **(E)** Stacked bar plot of species abundance at the genus level. **(F)** Differential microbiota at the phylum level. **(G)** Firmicutes/Bacteroidota ratio. **(H)** Differential microbiota at the genus level. **(I)** LEfSe differential analysis. **(J)** Abundance statistical chart for species function prediction. Data are presented as mean ± SD. Compared with the Control group, ^#^*P* < 0.05, ^##^*P* < 0.01, ^###^*P* < 0.001; compared with the Lop group, *P < 0.05, **P < 0.01, and ***P < 0.001; *n* = 6.

The effect of SHTB on gut microbiota dysbiosis in constipated mice was investigated using 16S rRNA sequencing. ASV-based clustering revealed 1,193 unique OTUs in the loperamide group and 1,023 in the control group, suggesting that constipation leads to changes in the number of OTUs compared to normal mice, and unique OTUs also appear after SHTB intervention (Figure 3A). Alpha diversity analysis revealed no significant differences in Ace, Chao1, and Simpson indices among the three groups (Figure 3C), possibly due to common ecological pressures or interfering factors in the experiment, such as dietary changes, medication use, or environmental factors. However, relative to the control group, the Lop group displayed divergent trends (either increases or decreases), whereas the SHTB intervention group exhibited a tendency toward normalization, suggesting a potential therapeutic effect. Beta diversity analysis showed a clear separation between the Lop group and the Control and SHTBH groups, indicating that constipation alters the structure of the gut microbiota in mice, and SHTB can regulate the composition and diversity of the gut microbiota in constipated mice, providing a protective effect against constipation (Figure 3B). Alpha diversity analysis revealed no significant differences in Ace and Simpson indices among the three groups (Figure 3C), possibly due to common ecological pressures or interfering factors in the experiment, such as dietary changes, medication use, or environmental factors. However, relative to the control group, the Lop group displayed divergent trends (either increases or decreases), whereas the SHTB intervention group exhibited a tendency toward normalization, suggesting a potential therapeutic effect. Differences at the phylum level showed that compared to the control group, the relative abundance of *Bacteroidota* decreased and *Desulfobacterota* increased in the Lop group, while SHTBH reversed these changes (Figures 3D–F). Furthermore, SHTB reversed the elevated *Firmicutes*/*Bacteroidota*ratio (F/B; Figure 3G). At the genus level, compared to the control group, the Lop group exhibited increased relative abundances of *Desulfovibrio, Enterorhabdus*, and *Streptococcus*, and decreased relative abundances of *Bifidobacterium, Ruminococcus*, and *Alistipes*, among others. Compared to the Lop group, the SHTBH group showed varying degrees of reversal (Figure 3H). LDA with a threshold score of 3 identified taxa with significantly different abundances among the three groups; higher LDA scores indicate a greater contribution of that taxon to the differences between groups. At the genus level, Desulfovibrio had a significant LDA score in the Lop group, indicating its unique role, consistent with the trend in species composition analysis; Bifidobacterium showed an increased LDA score in the SHTBH group, indicating its association with SHTB intervention (Figure 3I). Functional prediction analysis indicated that approximately 60% of the microbiota across all three groups were associated with intestinal metabolic functions (Figure 3J).

### 3.4 SHTB ameliorates tryptophan metabolic alterations in constipated mice

**Figure 4:**
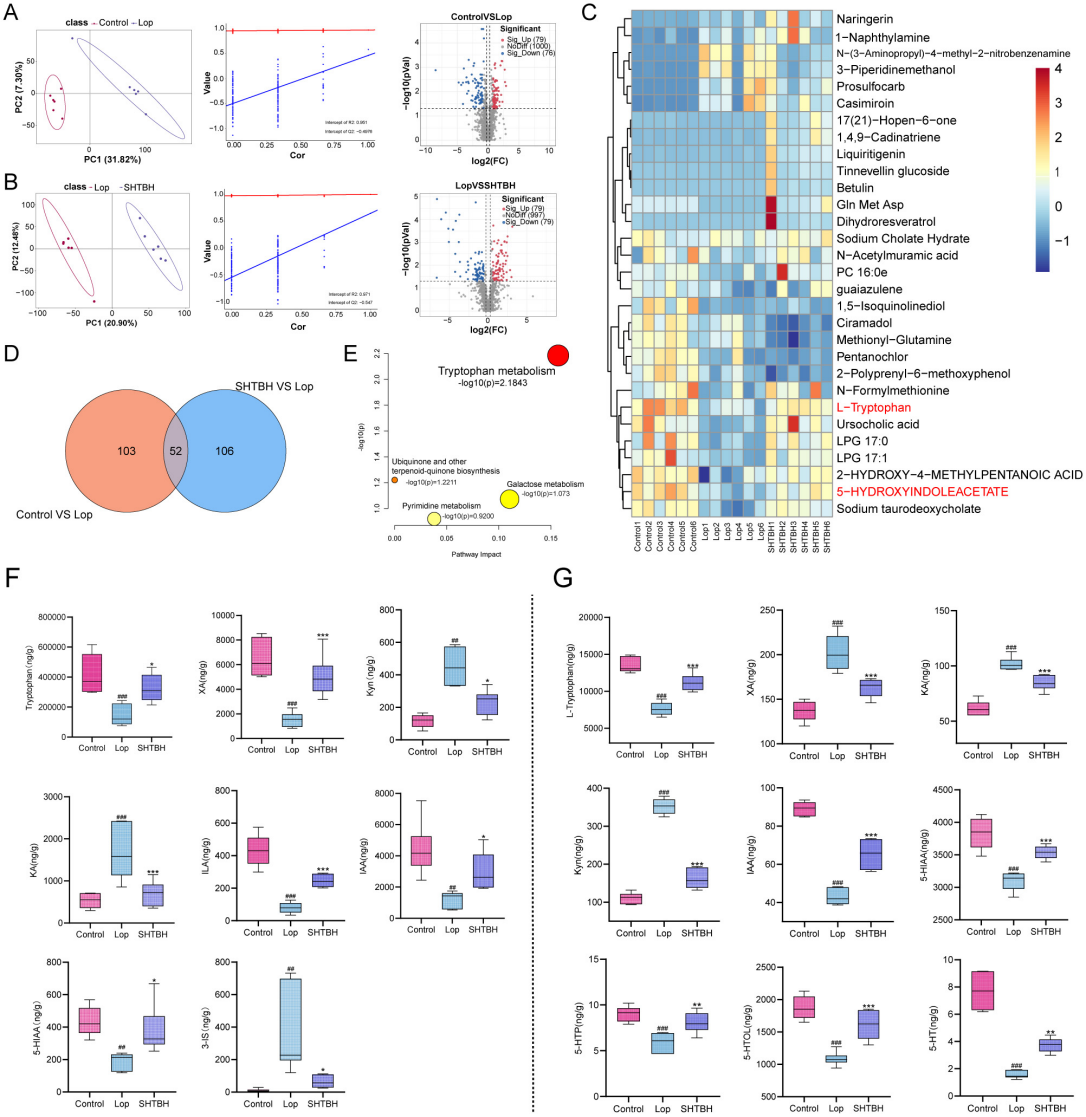
SHTB alleviates tryptophan metabolism disorder in constipated mice. **(A, B)** PLS-DA score plot, permutation test plot, volcano plot of differential metabolites. **(C)** Heatmap of differential metabolites across the three groups. **(D)** Venn diagram of differential metabolites between the three groups. **(E)** Metabolism pathway analysis diagram. **(F)** Tryptophan metabolites in feces. **(G)** Tryptophan metabolites in the colon. Data are presented as mean ± SD. Compared with the Control group, ^#^*P* < 0.05, ^##^*P* < 0.01, ^###^*P* < 0.001; compared with the Lop group, *P < 0.05, **P < 0.01, and ***P < 0.001; *n* = 6.

The impact of SHTB on stool metabolites in constipation mice was analyzed using non-targeted metabolomics. Partial least squares-discriminant analysis (PLS-DA) is a supervised multivariate method designed to maximize separation between groups. The PLS-DA model results for the control group vs. the Lop group (*R*^2^ = 0.951, *Q*^2^ > 0.5) and the SHTBH group vs. the Lop group (*R*^2^ = 0.971, *Q*^2^ > 0.5) showed no overfitting, and samples from each group exhibited significant separation (Figures 4A, B). In the comparative analysis between the Control group and the Lop group, 79 metabolites exhibited elevated levels while 76 showed reduced abundance; conversely, when contrasting the SHTBH cohort with the Lop group, we observed 79 upregulated and 79 downregulated metabolites. Pathway enrichment analysis was performed on the 52 shared differential metabolites. The results indicated that SHTB primarily modulated the following metabolic pathways: Tryptophan metabolism (–log10(p) = 2.1843), Ubiquinone and other terpenoid-quinone biosynthesis (–log10(p) = 1.2211), Galactose metabolism (–log10(p) = 1.073), and Pyrimidine metabolism (–log10(p) = 0.9200; Figures 4D, E). Among these, tryptophan metabolism exhibited the highest degree of enrichment. Notably, among the significantly altered metabolites across the three groups, the levels of L-Tryptophan and 5-HIAA were markedly reduced in the model group, and these reductions were reversed following SHTBH treatment (Figure 4C). These findings suggest that SHTB may alleviate constipation by ameliorating metabolic disorders in mice, with a particular emphasis on tryptophan metabolism.

Given the close relationship between tryptophan metabolism and constipation (Qin et al., 2024), we measured tryptophan metabolites in both fecal and colonic samples. Fecal tryptophan metabolite profiling revealed significant alterations in seven metabolites (Figure 4F). Compared with control group, loperamide-treated mice exhibited elevated levels of kynurenine pathway metabolites—XA, Kyn, and kynurenic acid (KA)—as well as 3-IS, alongside reduced levels of IAA and indole-3-lactic acid (ILA), and the serotonin metabolite 5-HIAA. SHTB treatment reversed these alterations. Colonic tryptophan metabolite analysis identified eight significantly altered metabolites (Figure 4G), including the five metabolites detected in feces (XA, Kyn, KA, IAA, 5-HIAA), with similar directional changes. Additionally, colonic levels of 5-hydroxytryptamine (5-HT), 5-hydroxytryptophan (5-HTP), and 5-hydroxytryptophol (5-HTOL) dropped in the Lop group and rose after SHTBH treatment (Figure 4G). Notably, both IAA and 5-HIAA exhibited significant alterations in feces and colon, suggesting their critical involvement in the effects of SHTB on constipation.

### 3.5 SHTB improves intestinal barrier function by activating AhR

**Figure 5:**
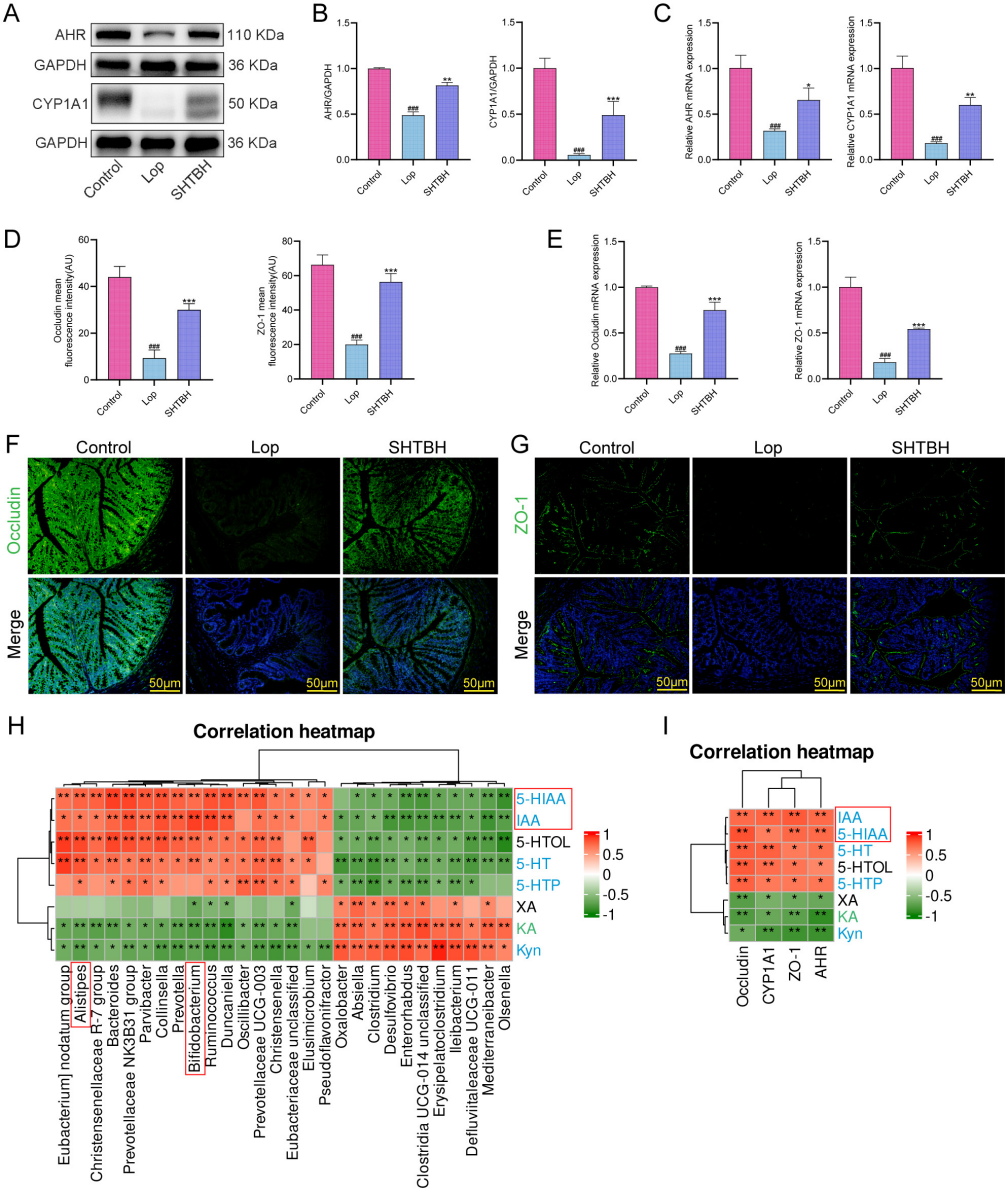
SHTB improves intestinal barrier function by activating AhR. **(A, B)** Expression levels of AhR and CYP1A1 proteins. **(C)** Expression of AhR and CYP1A1 mRNA. **(D, E)** Fluorescence intensity and mRNA expression of ZO-1 and Occludin. **(F, G)** Fluorescence images of ZO-1 and Occludin (Bar = 50 μm). **(H)** Correlation analysis between gut microbiota (genus) and colonic tryptophan. **(I)** Correlation analysis between colonic tryptophan and colonic AhR, CYP1A1, ZO-1, and Occludin. Data are presented as mean ± SD. Compared with the Control group, ^#^*P* < 0.05, ^##^*P* < 0.01, ^###^*P* < 0.001; compared with the Lop group, *P < 0.05, **P < 0.01, and ***P < 0.001; *n* =3-6.

Constipation compromises the architectural integrity of intestinal tight junctions, undermines barrier efficacy, and suppresses gut motility (Xie et al., 2026). Emerging evidence indicates that modulating tryptophan-derived indole metabolism represents a potent therapeutic approach for restoring epithelial barrier function and mitigating constipation symptoms (Ma et al., 2025). The above research confirms that SHTB can regulate various tryptophan indole metabolites, and indole derivatives are ligands of AhR. Intestinal homeostasis is preserved via the AhR signaling axis, which orchestrates the transcriptional regulation of tight junction proteins (TJPs) (Chi et al., 2025). As illustrated in Figures 5A–C, colonic tissues from the Lop group exhibited a marked suppression of AhR and its downstream target CYP1A1 relative to the Control group; notably, SHTBH treatment effectively abrogated this downregulation. Consistent with these findings, immunofluorescence staining and RT-qPCR analyses showed significantly reduced expression of ZO-1 and occludin in the Lop group compared with Control group. In contrast, SHTBH administration restored TJPs expression (Figures 5D–G), thereby re-establishing the continuous and intact architectural distribution of TJPs. This restorative effect stands in stark opposition to the fragmented and disorganized TJP patterning characteristic of the Lop group.

Spearman correlation analysis was used to examine the relationship between gut microbiota and colonic tryptophan metabolism. Beneficial bacteria, including *Bifidobacterium, Alistipes*, and *Lachnospiraceae NK4A136 group*, were positively correlated with colonic levels of IAA, 5-HIAA, 5-HT, and 5-HTOL, and negatively correlated with Kyn and KA. In contrast, potentially harmful bacteria such as *Desulfovibrio* and *Enterorhabdus* showed negative correlations with IAA, 5-HIAA, 5-HT, 5-HTP, and 5-HTOL, and positive correlations with Kyn, KA, and XA (Figure 5H). These results suggest that these bacterial genera are involved in the production of colonic tryptophan metabolites. Further analysis revealed that colonic expression levels of AhR, CYP1A1, ZO-1, and Occludin were significantly positively correlated with colonic IAA, 5-HIAA, 5-HT, 5-HTP, and 5-HTOL, and negatively correlated with colonic Kyn, KA, and XA (Figure 5I). Taken together, these findings indicate that SHTB may alleviate constipation by modulating AhR expression, repairing TJPs, and improving intestinal barrier function via microbial tryptophan metabolites.

### 3.6 SHTB activates AhR in a gut microbiota-dependent manner

**Figure 6:**
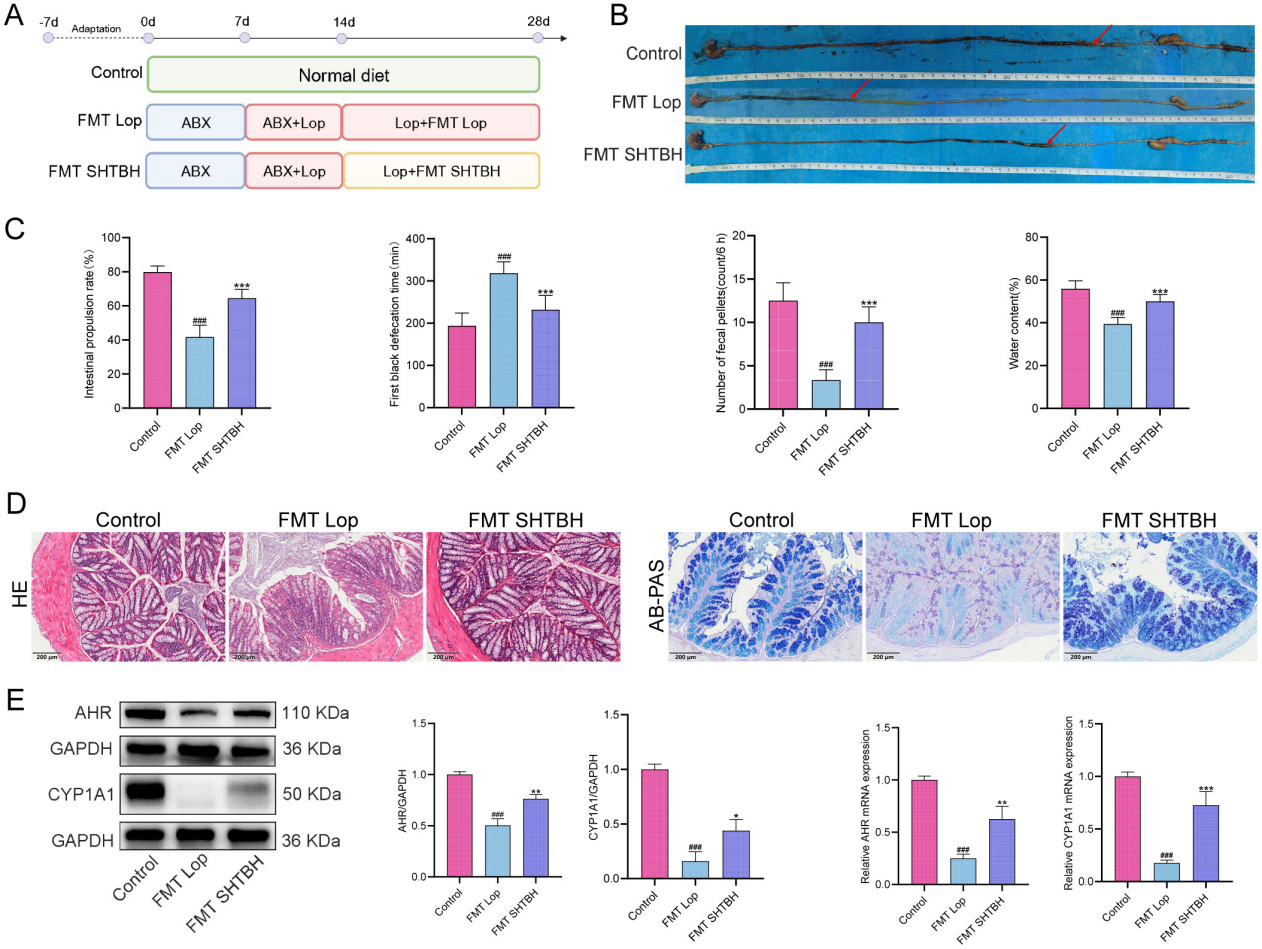
SHTB activates AhR in a gut microbiota-dependent manner. **(A)** Schematic diagram of the experimental design. **(B)** Representative images of intestinal propulsion. **(C)** Intestinal propulsion rate, First black stool defecation time, Number of fecal pellets expelled within 6 h, fecal water content. **(D)** HE staining of mouse colon and AB-PAS staining of mouse colon (Bar = 200 μm). **(E)** Protein expression and mRNA expression of AhR and CYP1A1. Compared with the Control group, ^#^*P* < 0.05, ^##^*P* < 0.01, ^###^*P* < 0.001; compared with the Lop group, *P < 0.05, **P < 0.01, and ***P < 0.001; *n* =3-6.

To further evaluate whether the gut microbiota regulated by SHTB is sufficient to produce therapeutic effects on constipation, we transplanted feces from SHTBH-treated constipated mice into a pseudo-germ-free constipated mouse model induced by Lop combined with ABX (Figure 6A). This treatment significantly accelerated gastrointestinal transit, evidenced by a reduced latency to first black stool expulsion, alongside substantial increases in the six-hour fecal pellet count, stool water content, and small intestinal propulsive rate (Figure 6B, C). Furthermore, compared to the FMT Lop group, the FMT SHTBH intervention markedly attenuated goblet cell depletion and mucin loss while mitigating histological injury in the colon (Figure 6D). In addition, SHTB fecal supplementation effectively restored the expression levels of AhR and CYP1A1 (Figure 6E). Therefore, FMT SHTBH exerted effects similar to those of direct SHTBH treatment in improving constipation, indicating that the intestinal microbiota mediated the beneficial effects of SHTB and activated AhR.

**Figure 7:**
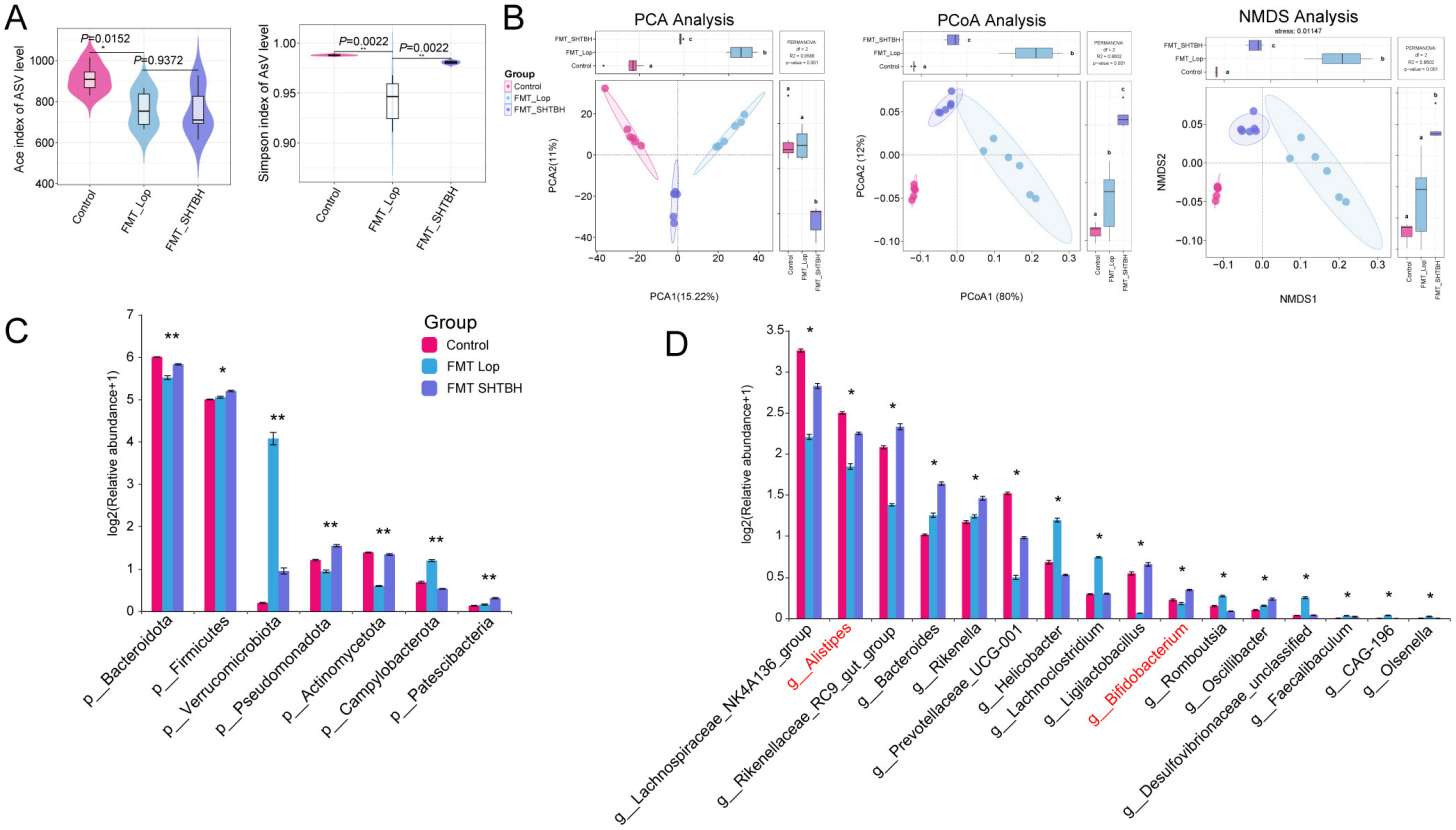
Analysis of gut microbiota after FMT. **(A)** Comparison of Alpha diversity indices (Ace, Simpson) among groups. **(B)** Beta diversity analysis based on PCA, PCoA, and NMDS. **(C)** Differential microbiota at the phylum level. **(D)** Differential microbiota at the genus level. Data are presented as mean ± SD. Compared with the FMT Control group, ^#^*P* < 0.05, ^##^*P* < 0.01, ^###^*P* < 0.001; compared with the FMT Lop group, *P < 0.05, **P < 0.01, and ***P < 0.001; *n* = 6.

Following FMT, 16S rRNA sequencing was repeated. The Ace index showed no significant difference, while the Simpson index revealed a significant difference between the FMT SHTBH and FMT Lop groups (Figure 7A). Moreover, multivariate analyses encompassing PCA, PCoA, and NMDS revealed a distinct segregation of the FMT Lop group from both the Control and FMT SHTBH groups, as illustrated in Figure 7B. Genus-level differential profiling revealed that, relative to the Control group, the FMT Lop group exhibited significantly diminished abundances of *Bifidobacterium, Alistipes, Rikenellaceae_RC9_gut_group*, and *Lachnospiraceae_NK4A136_group*, alongside a marked enrichment of Helicobacter and *unclassified Desulfovibrionaceae*. Conversely, the FMT SHTBH intervention partially reversed these dysbiotic shifts when compared directly with the FMT Lop group, indicating a restorative effect on microbial composition (Figures 7C, D). The above confirms that feces from mice in the SHTBH group, after FMT, partially reproduced key gut bacteria regulated by SHTB (such as *Bifidobacterium* and *Alistipes*), and these bacteria are closely related to tryptophan metabolism.

### 3.7 AhR is a key target for SHTB in improving constipation

**Figure 8:**
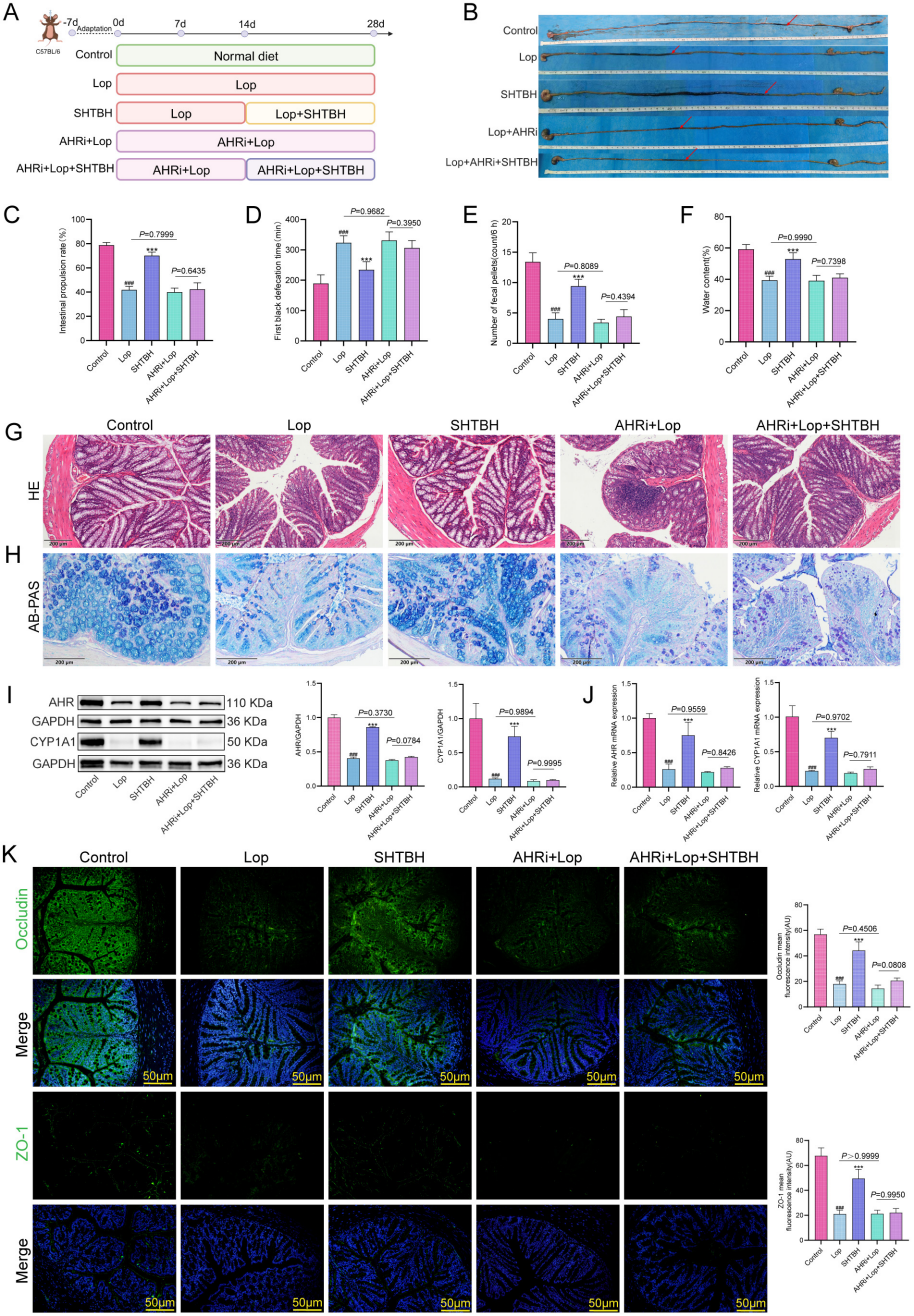
AhR is the key target for SHTB in improving constipation. **(A)** Schematic diagram of the experimental design. **(B)** Representative images of intestinal propulsion. **(C)** Intestinal propulsion rate; **(D)** first black stool defecation time. **(E)** Number of fecal pellets expelled within 6 h. **(F)** fecal water content. **(D, H)** H&E staining of mouse colon and AB-PAS staining of mouse colon (bar = 200 μm). **(I, J)** Protein expression and mRNA expression of AhR and CYP1A1. **(K)** Fluorescence intensity expression of ZO-1 and Occludin (Bar = 50 μm). Compared with the Control group, ^#^*P* < 0.05, ^##^*P* < 0.01, ^###^*P* < 0.001; compared with the Lop group, *P < 0.05, **P < 0.01, and ***P < 0.001; *n* =3-6.

Prior investigations indicate that SHTB potentially alleviates constipation through the modulation of microbial tryptophan metabolic pathways, subsequent activation of the AhR, and restoration of intestinal barrier integrity. To verify this hypothesis, we co-administered SHTB and an AhR inhibitor to constipated mice to elucidate the critical role of AhR in the efficacy of SHTB against constipation (Figure 8A). Our data demonstrate that pharmacological inhibition of AhR abrogated the beneficial effects of SHTB across multiple constipation-related parameters. Specifically, the antagonist reversed SHTB-mediated improvements in the latency to initial black stool expulsion, total fecal pellet output, stool hydration levels, and small intestinal transit velocity. Furthermore, this inhibition abrogated the protective effects on colonic histology, evidenced by the recurrence of tissue pathology and a significant reduction in goblet cell abundance (Figures 8B–H). Simultaneously, administration of the AhR antagonist markedly suppressed AhR and CYP1A1 expression within colonic tissues; notably, subsequent SHTB intervention failed to reverse this downregulation (Figures 8I, J). Immunofluorescence analysis substantiated that the capacity of SHTB to preserve intestinal barrier integrity is contingent upon AhR activation (Figure 8K). Taken together, these data demonstrate that the therapeutic efficacy of SHTB against constipation is predominantly driven by AhR signaling.

## 4 Discussion

The pathogenesis underlying constipation remains profoundly intricate and has not been completely clarified to date. Its clinical symptoms are refractory, and the overuse of laxatives is relatively common, yet therapeutic outcomes are often suboptimal. Traditional Chinese Medicine (TCM) offers distinct therapeutic advantages and is widely utilized in clinical settings. SHTB is a Chinese materia medica compound preparation developed based on TCM theory, with the effects of nourishing yin, replenishing qi, purging turbidity, and promoting bowel movements. To assess the therapeutic potential of SHTB, we established a murine constipation model via loperamide administration. Our findings indicate that SHTB treatment markedly reduced the latency to the first black stool defecation, elevated fecal moisture levels, and enhanced small intestinal transit efficiency. Furthermore, constipated mice exhibited compromised intestinal barrier integrity, characterized by a significant loss of goblet cells, reduced mucus secretion, and downregulated mucin expression in colonic tissue. SHTB administration effectively reversed these alterations.

Emerging evidence underscores gut microbiota dysbiosis as a pivotal driver in the pathogenesis of constipation, wherein intricate crosstalk between microbial communities and their metabolic byproducts critically modulates both the initiation and progression of this disorder. The gut microbiota of patients with constipation is primarily characterized by a relative decrease in beneficial microbial taxa, a relative increase in opportunistic pathogens, and a decline in species richness (Yang et al., 2022). In this study, alpha diversity was not significantly altered, but SHTB modulated the composition of the gut microbiota. The F/B ratio is a key marker of gut health, and constipation is associated with a higher F/B ratio (Yu et al., 2024). SHTB effectively regulates the abnormal F/B ratio and restores gut microbiota balance. Previous studies have shown that the abundances of *Bifidobacterium* and *Bacteroides* species in the feces of adult patients with functional constipation is significantly reduced (Kim et al., 2015). Genus-specific analysis revealed that, compared with the Control group, the Lop group exhibited decreased relative abundances of *Bifidobacterium, Alistipe*s, *Ruminococcus*, and *Lachnospiraceae_NK4A136_group*, accompanied by elevated levels of pathogenic bacteria such as *Desulfovibrio* and *Streptococcus*. Notably, the administration of SHTB reversed this trend.

Untargeted metabolomics studies indicate that tryptophan metabolism is the most significantly enriched pathway through which SHTB treats constipation. Tryptophan, a prototypical aromatic amino acid, undergoes metabolic conversion predominantly through three principal routes: the kynurenine pathway, the serotonin biosynthetic cascade, and the indole derivative pathway. Accumulating evidence indicates that the gut microbiome not only modulates the initial two metabolic pathways but also exerts a direct regulatory effect on the third pathway (Klaessens et al., 2022). Research shows that individuals with constipation have higher levels of homovanillic acid (HVA), and lower levels of 5-HIAA (Chojnacki et al., 2024). Tryptophan can be converted to indole by tryptophanase, an enzyme produced by various gut microbes, an enzyme widely distributed in various microorganisms, including *Escherichia coli, Clostridium, Bacteroides*, and *Lactobacillus* (Agus et al., 2018). *Bifidobacterium* can induce the conversion of Trp to IAA to ameliorate hepatic steatosis and inflammation (Min et al., 2024). Recent studies have also confirmed that *Bifidobacterium animalis subsp. lactis A6* can influence tryptophan metabolism by modulating the gut microbiota (such as increasing the abundance of *Bifidobacterium* and *Lactobacillus*), specifically by upregulating TPH1 expression to promote 5-HT secretion while inhibiting Kyn production, ultimately alleviating constipation and depressive symptoms (Wang et al., 2025). This study showed that after SHTB treatment, beneficial bacteria such as *Bifidobacterium* and *Alistipes* increased, while the levels of tryptophan metabolites IAA, 5-HIAA, and ILA increased, and the levels of XA, Kyn, KA, and 3-IS decreased.

It has been reported that the postbiotic Probio-Eco, by specifically mediating the Trp-5-HTP-5-HT pathway and the Trp-3-indoleacrylic acid pathway, drives the AhR axis, regulates the expression of colonic transport-related genes (MUC2, AQP3, AQP4), synergistically improves intestinal motility, and effectively alleviates constipation (Ma et al., 2025). Recent studies have shown that *Alistipes onderdonkii* and *Bifidobacterium adolescentis CCFM8630* can promote AhR expression, increase TJPs expression, enhance the colonic barrier, and significantly improve colonic environmental health through metabolites (such as IAA, 5-HIAA, and KA) generated via the Trp-indole or Trp-Kyn pathways (Zhong et al., 2024; Zhang et al., 2025). This study indicates that SHTB can promote the expression of AhR, CYP1A1, ZO-1, and Occludin in the colon. Correlation analysis showed that beneficial bacteria such as *Bifidobacterium* and *Alistipes* exhibited positive correlations with colonic indole metabolites IAA, as well as effector molecules AhR, CYP1A1, ZO-1, and Occludin. To validate the critical role of the gut microbiota in mediating the therapeutic efficacy of SHTB, we performed fecal microbiota transplantation experiments. Notably, recipients colonized with microbiota derived from SHTB-treated donors exhibited clinical improvements analogous to those observed following direct SHTB intervention, thereby confirming that the microbiota serves as a critical effector of SHTB's mechanism of action. These results not only demonstrated the restoration of the abundance of key gut microbiota regulated by SHTB (such as *Bifidobacterium* and *Alistipes*) and the expression levels of AhR and CYP1A1, but also confirmed that SHTB's effects on repairing the intestinal barrier and improving constipation are mediated by the gut microbiota. Additionally, applying an AhR antagonist to block AhR in constipated mice counteracted the improvements in constipation-associated pathological features and molecular indicators induced by SHTB. In summary, SHTB may regulate tryptophan metabolism in a gut microbiota-dependent manner, activate AhR, and improve the intestinal barrier to alleviate constipation.

Although this study provides strong evidence that the gut microbiota and its tryptophan metabolites are pivotal mediators in the anti-constipation effect of SHTB, certain limitations persist. IAA, 5-HIAA, and other important tryptophan metabolites have not been validated for efficacy through individual administration, while the functions of other metabolites regulated by SHTB remain unclear. Furthermore, the production of tryptophan metabolites likely entails the synergistic action of multiple bacteria. Although this study confirms the regulatory effect of SHTB on the gut microbiota, further research is needed to identify specific bacterial species capable of producing tryptophan metabolites such as IAA. Identifying these species is crucial for a comprehensive understanding of the role of the gut microbiota in the anti-constipation mechanism of SHTB.

## 5 Conclusion

**Figure 9:**
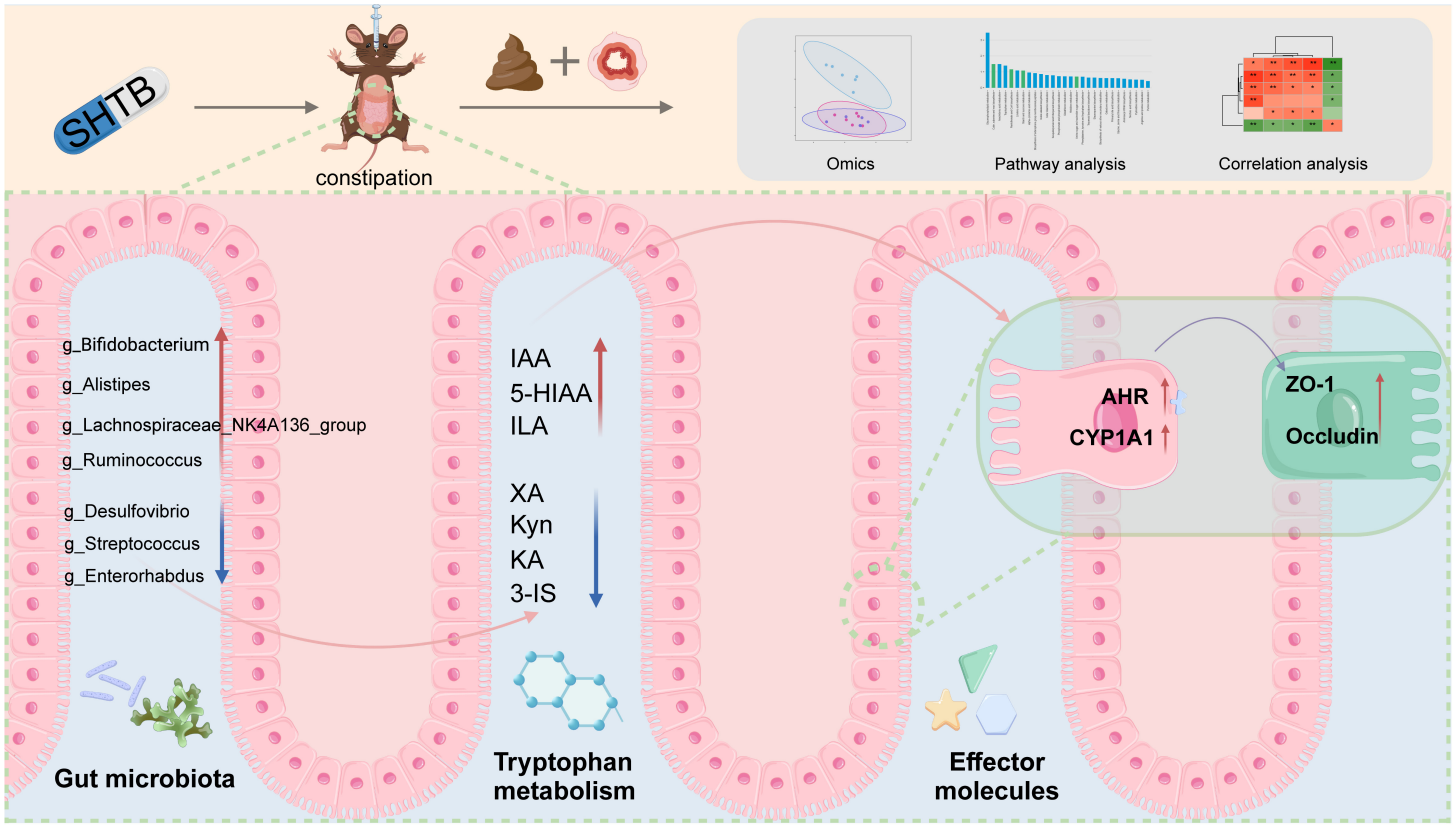
SHTB improves constipation symptoms by enriching beneficial bacteria such as *Bifidobacterium* and *Alistipes*, enhancing the production of tryptophan metabolites (e.g., IAA, 5-HIAA), thereby activating AhR and improving intestinal barrier integrity.

Our findings indicate that SHTB alleviates constipation by augmenting populations of beneficial microbes, specifically *Bifidobacterium* and *Alistipes*, thereby stimulating the biosynthesis of tryptophan-derived metabolites such as IAA and 5-HIAA. This metabolic shift triggers AhR signaling, which reinforces intestinal barrier function and ultimately mitigates constipation symptoms (Figure 9). Elucidating the “SHTB-gut microbiota-Trp-AhR” signaling axis uncovers the specific microbial mechanisms through which TCM ameliorates constipation, thereby establishing a robust theoretical foundation for the future design of innovative TCM formulations dedicated to the prevention and management of this condition.

## Data Availability

The data presented in the study are deposited in the NCBI (https://www.ncbi.nlm.nih.gov/bioproject/), with the accession numbers PRJNA1444881 and PRJNA1471448. Additional data supporting the findings of this study are available from the corresponding author upon reasonable request.
