# Supplementary material for: Shouhui Tongbian Capsules alleviate constipation by modulating the gut microbiota–tryptophan metabolism–AhR signaling axis: a mechanistic study
Source: Front Microbiol. 2026 Jun 30;17:1844612. doi: 10.3389/fmicb.2026.1844612 (PMC13365304; doi:10.3389/fmicb.2026.1844612)
Supplement: Supplementary file 1 [file Supplementary_file_1.DOCX]

Supplementary Material

**Table S1. Drug information**

| Material Name | Manufacturer/Source | batch number |
| --- | --- | --- |
| Dry extract of Shouhui Tongbian capsule | Lunan Houpu Pharmaceutical Co., Ltd (Linyin, Shandong, China) | 26220161 |
| *Aloe vera* (L.) Burm.f. | Zhijia Pharmaceutical Co., Ltd. (Hebei, China) | 220401 |
| *Citrus × aurantium* L. |  | 221201 |
| *Lycium chinense* Mill. |  | 240101 |
| *Senna tora* (L.) Roxb. |  | 241017 |
| *Atractylodes macrocephala* Koidz. | Shandong Dingshun Chinese Medicine Decoction Co., Ltd (Shandong, China) | 20200827001 |
| *Reynoutria multiflora* (Thunb.) Moldenke | Shao Hua Tang Sinopharm Co., Ltd (Anhui, China) | 1240422 |
| *Colla Corii* Asini | Dong-E-E-Jiao Co., Ltd (Liaocheng, Shandong, China) | 2103010 |
| *Panax ginseng* C.A.Mey. | Anhui Hansheng Pharmaceutical Co., Ltd (Anhui, China) | YL530-20230503 |

**Methods：**

**S2. UPLC-Q-Orbitrap-MS Analysis**

The seven botanical drugs were processed via an ethanol-based extraction protocol. Specifically, 10 g of each botanical material was accurately weighed and combined with 75% ethanol at a solid-to-liquid ratio ranging from 1:10 to 1:12 (w/v). This mixture underwent three successive extraction cycles, each lasting 1 hour at 90°C. Following every cycle, the suspension was filtered to isolate the supernatant. All collected filtrates were pooled and concentrated using a rotary evaporator (model N-1300; EYELA, Japan) operated at 55°C under a reduced pressure of 0.08 MPa. The final concentrate was subsequently lyophilized to yield a dry powder, which was preserved at −20°C for subsequent applications.

Weigh 0.2 grams of SHTB powder, mix it with 1.0 milliliters of 80% methanol, then grind, vortex, and centrifuge to collect the supernatant. Analyze the active components of SHTB using an ultra-high performance liquid chromatography system (UltiMate 3000 RS, Thermo, USA) and a Q Exactive Orbitrap mass spectrometer (QE, Thermo, USA). Chromatographic separation was performed using an AQ C18 column (2.1 mm × 150 mm, 1.8 μm, Welch, USA). Data were processed using CD3.3 software and matched and retrieved using the mzCloud database.

Chromatographic separation was achieved on a Welch AQ-C18 column (150 × 2.1 mm, 1.8 μm) at 35 °C, with the autosampler at 10.0 °C and injection volume of 10 μL. The mobile phase was 0.1% formic acid aqueous solution (A) and acetonitrile (B) at a flow rate of 0.7 mL/min, using gradient elution: 98%A (2%B) at 1 min, 80%A (20%B) at 5 min, 50%A (50%B) at 10 min, 20%A (80%B) at 15 min, 5%A (95%B) at 20 min (held to 27 min), back to 98%A at 28 min, and equilibrated to 30 min. Mass spectrometry was performed on a Q Exactive high-resolution mass spectrometer (Thermo Fisher) with an ESI source in positive/negative ion switching mode (Full mass/dd-MS2). The resolution was 70000 (full mass) and 17500 (dd-MS2) over m/z 100.0–1500.0. ESI voltage was 3.2 kV, capillary temperature 300 °C; high-purity Ar (≥99.999%) was collision gas (CE: 30, 40, 60 N), high-purity N2 (≥99.999%) as sheath gas (40 Arb) and auxiliary gas (15 Arb, 350 °C). Data acquisition lasted 30 min, with data preprocessed via Compound Discoverer 3.3 and matched against the mzCloud database.

**Table S3 The Primer sequences used in RT-qPCR**

| Species | Gene | Forward primer(5’-3’) | Reverse primer(5’-3’) |
| --- | --- | --- | --- |
| Mouse | AhR | AGCCGGTGCAGAAAACAGTAA | AGGCGGTCTAACTCTGTGTTC |
| Mouse | CYP1A1 | GGGTTTGACACAGTCACAACT | GGGACGAAGGATGAATGCCG |
| Mouse | ZO-1 | GCCGCTAAGAGCACAGCAA | TCCCCACTCTGAAAATGAGGA |
| Mouse | Occludin | GTCCACCTCCTTACAGACCT | CTGGCTGAGAGAGCATCGG |

**Methods：**

**S4. Analysis of Gut Microbiota Through 16S rDNA Gene Sequencing**

Total microbial DNA was extracted from fecal samples using a fecal genomic DNA extraction kit (AU46111-96, BioTeke, China) according to the manufacturer's protocol. DNA concentration was quantified using a Qubit fluorometer (Invitrogen, USA). PCR amplification was performed with universal primers 341F/805R (341F: 5′-CCTACGGGNGGCWGCAG-3′; 805R: 5′-GACTACHVGGGTATCTAATCC-3′) under the following conditions: initial denaturation at 98°C for 30 s, followed by 32 cycles of denaturation at 98°C for 10 s, annealing at 54°C for 30 s, and extension at 72°C for 45 s, with a final extension at 72°C for 10 min. PCR products were purified using AMPure XT beads (Beckman Coulter Genomics, USA) and quantified by Qubit. Qualified PCR products were verified using an Agilent 2100 Bioanalyzer (Agilent, USA) and the Illumina Library Quantification Kit (Kapa Biosciences, USA), followed by sequencing analysis on the Illumina NovaSeq 6000 platform (PE250) by LC-Bio Technology Co., Ltd. (Hangzhou, China).

**S5. Fecal Untargeted Metabolomics**

Collected fecal samples were thawed on ice, and metabolites were extracted with precooled 80% methanol. Briefly, 50 mg of sample was mixed with 0.5 mL 80% precooled methanol, stored at -20°C for 30 min, centrifuged at 20,000 g for 15 min. The supernatant was collected, vacuum-dried, redissolved in 100 μL 80% methanol, and stored at -80°C until LC-MS analysis. Pooled QC samples were prepared by mixing 10 μL of each extraction mixture, and all samples were analyzed by LC-MS in sequence.Chromatographic separation was performed on an UltiMate 3000 UPLC System (ThermoFisher Scientific, Bremen, Germany) with an ACQUITY UPLC T3 column (100 mm×2.1 mm, 1.8 μm, Waters, Milford, USA) at 40°C. The mobile phase consisted of solvent A (5 mM ammonium acetate + 5 mM acetic acid) and solvent B (acetonitrile) at a flow rate of 0.3 mL/min, with gradient elution: 0–0.8 min (2% B), 0.8–2.8 min (2%–70% B), 2.8–5.6 min (70%–90% B), 5.6–6.4 min (90%–100% B), 6.4–8.0 min (100% B), 8.0–8.1 min (100%–2% B), 8.1–10 min (2% B).Metabolites were detected by a Q-Exactive high-resolution tandem mass spectrometer (Thermo Scientific) in both positive and negative ion modes. Precursor spectra (70–1050 m/z) were acquired at 70,000 resolution (AGC target: 3e6, max inject time: 100 ms); DDA mode (top 3) was used, with fragment spectra collected at 17,500 resolution (AGC target: 1e5, max inject time: 80 ms). A QC sample was analyzed every 10 samples to evaluate LC-MS stability.Statistical analysis was performed in R 4.0.0: raw intensities were normalized by "medium" method; hierarchical clustering (pheatmap), PCA (metaX), PLSDA (ropls, with VIP calculation) and Pearson correlation analysis (cor package) were conducted. Differential metabolites were screened by T-test (P < 0.05, fold change > 1.2) and VIP value. KEGG pathway enrichment analysis (hypergeometric test) and GSEA (v4.1.0, MSigDB, |NES| > 1, NOM p-val < 0.05, FDR q-val < 0.25) were performed, and network maps were constructed based on metabolite-related pathways.

**S6. Fecal and Colon Targeting Tryptophan Metabolism**

LC-MS grade acetonitrile (ACN), methanol (MeOH), formic acid, ammonium acetate, raw water, and all standards were purchased from CNW (Shanghai, China), WATSON, Sigma-Aldrich (St. Louis, MO, USA), ANPEL (Shanghai, China), and ALTER (Tianjin, China), respectively.Approximately 50 mg of sample was accurately weighed, mixed with 500 μL pre-cooled (-80 ℃ overnight) 80% methanol-water for protein precipitation, ground with two steel beads, vortex-extracted for 20 min, and centrifuged (20000 rcf, 4 ℃) for 15 min. The supernatant was collected, freeze-dried, re-dissolved in 100 μL 5 mmol/L ammonium acetate solution (containing 0.01% formic acid) + ACN (95:5, v/v), and the supernatant was subjected to LC-MS/MS analysis.Target compounds were separated and quantified using an AB Sciex JasperTM μLtra performance liquid chromatograph coupled with an AB SCIEX 4500MD triple quadrupole mass spectrometer. Chromatographic separation was performed on an Agilent Poroshell 120 EC-C18 column (3.0×150 mm, 2.7 μm) with mobile phases A (5 mmol/L ammonium acetate aqueous solution containing 0.01% formic acid) and B (ACN); the injection volume was 5 μL, and column temperature was 40 ℃.ESI-MS/MS was operated in both positive and negative ion modes (ion source: turbo spray; temperature: 400°C; ion spray voltage: 4500 V for positive, -4500 V for negative; curtain gas: 30.0 psi), with MRM transitions and collision energy (CE) optimized. Qualification was based on retention time and MRM fragment ions (vs. standards), and quantification was performed by external standard method with calibration curves (standard concentration as abscissa, peak area as ordinate). Samples exceeding the calibration curve range were diluted and retested.Missing values (below limit of quantification) were imputed as 0. Principal component analysis, metabolite correlation analysis, hierarchical clustering analysis, t-test, and orthogonal partial least squares discriminant analysis (OPLS-DA) were performed. Differential metabolites were defined as those with p-value < 0.05, fold change > 1, and VIP > 0, which were further subjected to KEGG and network enrichment analyses.

**Table S7. Analysis of chemical constituents of Shouhui Tongbian capsule based on UHPLC-Q-Exactive-MS**

| Name | Formula | ppm | m/z | RT [min] | Reference Ion |
| --- | --- | --- | --- | --- | --- |
| Meranzin hydrate | C15 H18 O5 | -2.68 | 261.1114 | 10.287 | [M+H-H2O]+1 |
| Genistein | C15 H10 O5 | -0.84 | 269.0453 | 15.025 | [M-H]-1 |
| Zapotin | C19 H18 O6 | -1.96 | 343.1169 | 13.764 | [M+H]+1 |
| L-Isoleucine | C6 H13 N O2 | 0.41 | 132.102 | 2.222 | [M+H]+1 |
| Aloenin | C19 H22 O10 | -2.26 | 411.1271 | 8.837 | [M+H]+1 |
| Trioxsalen | C14 H12 O3 | -1.47 | 261.1118 | 14.612 | [M+H+MeOH]+1 |
| α-Lapachone | C15 H14 O3 | -1.04 | 243.1013 | 15.543 | [M+H]+1 |
| Bergapten | C12 H8 O4 | -1.01 | 217.0493 | 12.834 | [M+H]+1 |
| (3R)-8-hydroxy-3-(4-hydroxyphenyl)-3,4-dihydro-1H-2-benzopyran-1-one | C15 H12 O4 | -2.5 | 239.0696 | 9.935 | [M+H-H2O]+1 |
| (2E)-3-(3,4-dimethoxyphenyl)prop-2-enoic acid | C11 H12 O4 | -1.37 | 209.0806 | 13.128 | [M+H]+1 |
| Auraptene | C19 H22 O3 | -0.98 | 299.1639 | 17.822 | [M+H]+1 |
| Arachidonic acid | C20 H32 O2 | 0.14 | 303.233 | 19.623 | [M-H]-1 |
| Griseoxanthone C | C15 H12 O5 | -1.7 | 273.0753 | 10.737 | [M+H]+1 |
| scopoletin | C10 H8 O4 | -3.2 | 193.0491 | 9.223 | [M+H]+1 |
| biochanin A | C16 H12 O5 | 0.07 | 283.0613 | 17.094 | [M-H]-1 |
| L-Tyrosine | C9 H11 N O3 | -0.83 | 182.081 | 1.456 | [M+H]+1 |
| 5,6,2'-Trimethoxyflavone | C18 H16 O5 | -0.97 | 313.1067 | 13.569 | [M+H]+1 |
| Daidzein | C15 H10 O4 | -0.73 | 253.0505 | 16.392 | [M-H]-1 |
| Marmesin | C14 H14 O4 | -2.85 | 247.0958 | 9.415 | [M+H]+1 |
| 3-(propan-2-yl)-octahydropyrrolo[1,2-a]pyrazine-1,4-dione | C10 H16 N2 O2 | 0.9 | 197.1286 | 7.11 | [M+H]+1 |
| Cryptotanshinone | C19 H20 O3 | -0.72 | 297.1483 | 16.417 | [M+H]+1 |
| Stearolic acid | C18 H32 O2 | -0.93 | 263.2367 | 14.218 | [M+H-H2O]+1 |
| formononetin | C16 H12 O4 | -0.55 | 269.0807 | 15.016 | [M+H]+1 |
